# The LTB4-BLT1 axis attenuates influenza-induced lung inflammation by suppressing NLRP3 activation

**DOI:** 10.1038/s41420-025-02450-8

**Published:** 2025-04-06

**Authors:** Cheng Wei, Yitian Xu, Ying Zheng, Lizhe Hong, Chen Lyu, Haibo Li, Bin Cao

**Affiliations:** 1 Peking University China-Japan Friendship School of Clinical Medicine, Beijing, China; 2 National Center for Respiratory Medicine; State Key Laboratory of Respiratory Health and Multimorbidity; National Clinical Research Center for Respiratory Diseases; Institute of Respiratory Medicine, Chinese Academy of Medical Sciences; Department of Pulmonary and Critical Care Medicine, Center of Respiratory Medicine, China-Japan Friendship Hospital, Beijing, China; 3 Chinese Academy of Medical Sciences and Peking Union Medical College, Beijing, China; 4 Department of Pulmonary and Critical Care Medicine, China-Japan Friendship Hospital, Capital Medical University, Beijing, China; 5 New Cornerstone Science Laboratory, Department of Pulmonary and Critical Care Medicine, Center of Respiratory Medicine, China-Japan Friendship Hospital, Beijing, China

**Keywords:** Microbiology, Influenza virus

## Abstract

The mortality associated with influenza A virus (IAV) infection typically results from excessive immune responses, leading to immunopathological lung damage and compromised pulmonary function. Various immunomodulators are seen beneficial when used in conjunction with direct anti-infection treatment. Leukotriene B4 (LTB4) is a derivative of arachidonic acid (AA) and has been shown to be advantageous for numerous infectious diseases, allergies, and autoimmune disorders. Nonetheless, the function of LTB4 in influenza infection remains unclear. This study demonstrates that LTB4 and its primary receptor BLT1, as opposed to the secondary receptor BLT2, act as a protective immune modulator during influenza infection in bone marrow-derived macrophages and mouse models. Mechanistically, LTB4 promotes K27-linked and K48-linked polyubiquitination of the NLRP3 protein at its K886 and K1023 sites via a cAMP/PKA-dependent pathway, which inhibits NLRP3 inflammasome assembly and thereby diminishes subsequent NLRP3 inflammasome activation. The consequent decline in the release of IL-1β and IL-18 leads to a reduction in inflammation caused by viral infection. Furthermore, the administration of a LTB4 treatment in a fatal IAV infection model can mitigate the excessive NLRP3 inflammasome activation and reduce IAV-induced severe pulmonary damage. These findings illustrate the protective function of LTB4 in fatal IAV infection by mitigating the severe inflammation induced by the virus.

## Introduction

An illness caused by the influenza A virus (IAV) continues to pose a considerable global health concern and economic burden. The Global Burden of Disease (GBD) Study predicts that IAV accounts for over 145,000 deaths annually across all age demographics [1]. Clinical data from our previous study has demonstrated that critically ill patients infected with IAV commonly manifest acute lung injury (ALI) [2, 3]. Additionally, several studies have also demonstrated that the intensity and course of influenza infection are associated with virus-induced tissue damage and increased release of inflammatory cytokines [4–6]. Thus, the incorporation of immunomodulators alongside antiviral therapy, as opposed to antiviral treatment in isolation, has gained prominence in alleviating the adverse effects of severe influenza pneumonia [4, 6–10]. The NLRP3 inflammasome, an essential element of the innate immune system, is a multiprotein complex consisting of NLRP3, apoptosis-associated speck-like protein with a caspase recruitment domain (ASC), and pro-caspase-1 [11]. Exogenous pathogenic invasions and endogenous cellular damage can trigger NLRP3 inflammasome assembly, resulting in caspase-1 activation and the subsequent maturation and release of IL-1β and IL-18 [12]. The NLRP3 inflammasome activation serves as a protective function by triggering innate and adaptive immune responses against IAV infection [13–15]. Recent studies have emphasized its bidirectional and intricate significance; notably, the over-activation of the NLRP3 inflammasome leads to pulmonary immunopathological damage, a critical factor in IAV-induced systemic hyperinflammation and immunological microenvironment dysregulation [13–15]. Our group and others have documented that over-activation of NLRP3 inflammasome intensifies immunopathological damage and pulmonary inflammation during IAV infection [4, 16]. MCC950, a targeted inhibitor of NLRP3, has demonstrated considerable protection against fatal influenza in mice by diminishing proinflammatory cytokine levels, including IL-1β and IL-18, in bronchoalveolar lavage fluid (BALF) and mitigating pulmonary immunopathological damage [17]. Lipid mediator leukotriene B4 (LTB4), a lipid mediator synthesized from arachidonic acid (AA), participates in various critical biological processes in reaction to stimuli [18]. Comprehensive studies have demonstrated that the LTB4-BLT1 (leukotriene B4 receptor 1) axis is involved in multiple acute and chronic inflammatory conditions, encompassing infectious illnesses, allergies, and autoimmune disorders [18, 19]. Recent studies indicate that LTB4 serves as a chemotactic factor in response to infections and also regulates immune cell function, especially in macrophages, by demonstrating anti-inflammatory effects and improving disease resilience [20–24].

For example, acts as an antagonistic agent to prostaglandin E2 (PGE2), a recognized proinflammatory mediator, in multiple disease models [25, 26]. Nonetheless, the exact function of LTB4 in influenza virus infection remains little elucidated. The modulation of NLRP3 inflammasome activation by LTB4 has been documented in many in vivo and in vitro models under diverse stimulation conditions [20, 27, 28]. The effects of LTB4 differ under various stimulation settings, perhaps resulting in completely opposing consequences [20, 27]. Nonetheless, the influence of LTB4 on NLRP3 inflammasome activation specifically triggered by IAV infection remains predominantly unexamined. This study presents the protective function of the LTB4-BLT1 axis in IAV infection, both in vitro and in vivo, facilitated via an inflammasome-dependent mechanism.

Our data indicate that the activation of the LTB4-BLT1 axis markedly diminishes the overproduction of proinflammatory cytokines, mitigates acute lung injury, and eventually enhances survival in a deadly influenza A virus mouse model. These findings underscore the therapeutic potential of targeting the LTB4-BLT1 axis to address severe influenza pneumonia.

## Results

### LTB4/BLT1 axis attenuates IAV-induced pulmonary immune pathology in mice

**Fig. 1:**
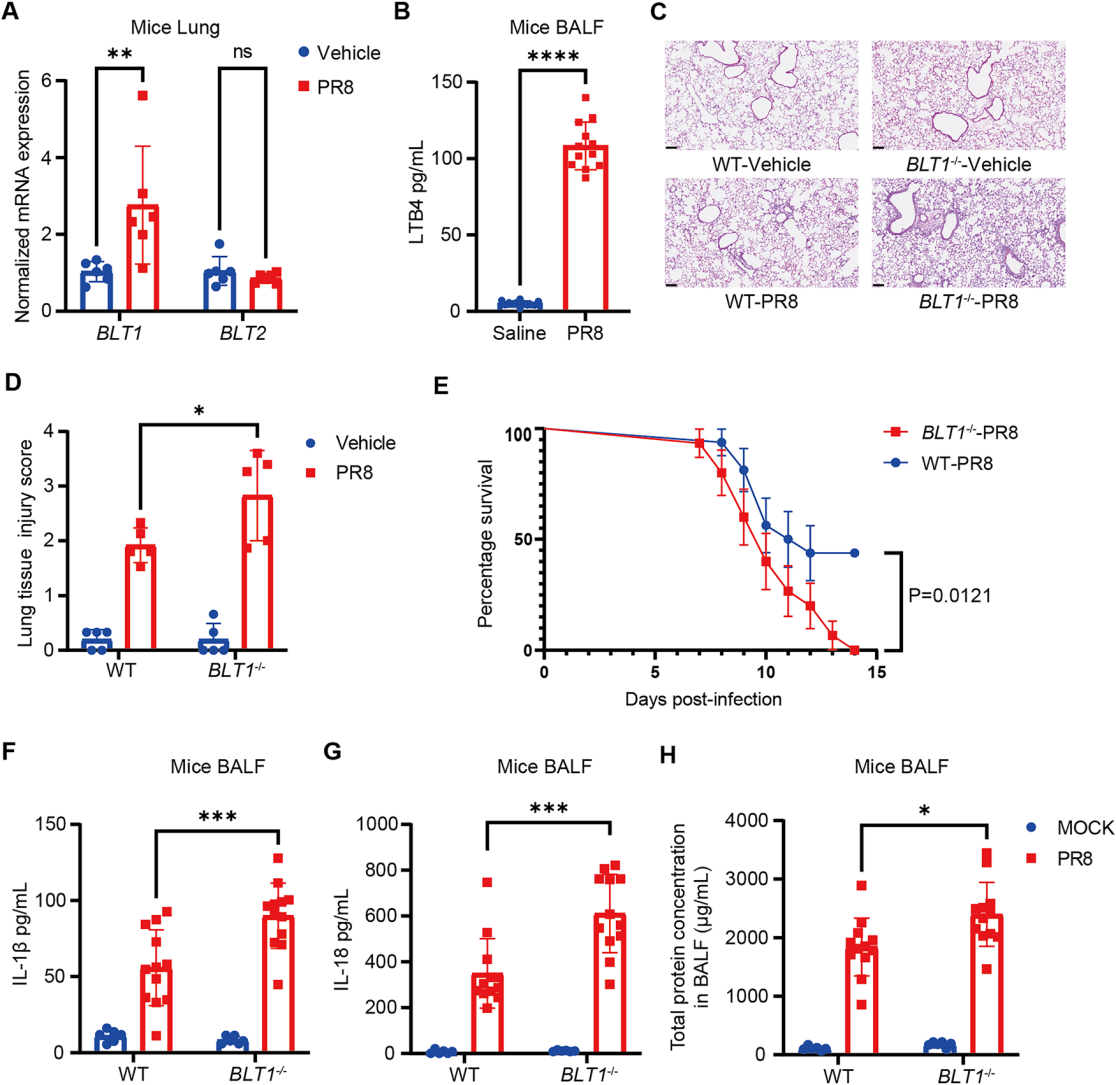
LTB4/BLT1 axis attenuates IAV-induced pulmonary immune pathology in mice. **A** WT mice were infected with an LD50 dose (5,000 pfu) of IAV. *BLT1* and *BLT2* expression in the lungs at 5 days post-infection was determined by reverse transcription qPCR *(BLT1*, *n* = 6 mice; *BLT2*, *n* = 6 mice). (representative of three assays). **B** WT mice were infected with IAV (5000 pfu), and LTB4 levels in the BALF were assessed by ELISA at 5 days post-infection (*n* = 12 mice). (representative of three assays). **C** Micrographs of haematoxylin and eosin-stained lung sections prepared at 5 days after IAV infection. Scale bars represent 100 µm. (representative of three assays). **D** Semiquantitative histological scoring of lung injury, and the experiment was conducted in a blinded manner. **E**, WT and *BLT1*^*−/−*^ mice were infected with an LD50 dose (5000 pfu) of IAV, and the survival was monitored (WT, *n* = 16; *BLT1*^*−/−*^, *n* = 15 mice). (representative of three assays). IL-1β (**F**), IL-18 (**G**) and total protein (**H**) levels in the BALF (uninfected, *n* = 6; IAV, *n* = 12 mice per group) at day 6 post-infection, measured by ELISA or BCA, respectively. (representative of three assays). Statistics: mean ± SD; unpaired, two-tailed Student’s *t* test (**A**, **B**); one-way ANOVA (**F**–**H**), or two-sided log-rank test (**E**); *p < 0.05, **p < 0.01, ***p < 0.001 and ****p < 0.0001.

To examine the function of the LTB4/BLT1 axis in IAV infection, we initially evaluated LTB4 production in vivo utilizing a non-lethal mouse model of IAV infection (LD50; 5,000 pfu). The expression of *BLT1*, the primary receptor for LTB4, was significantly upregulated in lung tissue after IAV infection (Fig. 1A), while there was no statistically significant difference in the expression level of *BLT2*, which was the secondary receptor for LTB4 (Fig. 1A). Furthermore, a significant elevation in LTB4 levels was seen in the bronchoalveolar lavage fluid (BALF) of IAV-infected mice (Fig. 1B). To confirm the importance of LTB4/BLT1 axis in IAV infection, we challenged the *BLT1*-deficient (*BLT1*^−/−^) mice with the same dose of IAV. *BLT1*^*−/−*^ mice were more susceptible to IAV compared to wild type (WT) mice (Fig. 1C–E). *BLT1*^−/−^ mice exhibited significantly greater lung inflammatory infiltration and elevated pathological damage scores (Fig. 1C, D). Furthermore, the survival rate in the *BLT1*^−*/−*^ group was markedly worse to that of the control group (Fig. 1E), consistent with others research [23, 24]. Notably, *BLT1* deletion (KO) significantly altered cytokine secretion in the BAlF. On day 6 post-infection, we performed Enzyme-linked immunosorbent assay (ELISA) and observed higher levels of IL-1β (Fig. 1F) and IL-18 (Fig. 1G) in BALF compared to the control group, respectively. Additionally, the total protein concentration, a hallmark of the alveolo-capillary membrane impairment induced by IAV infection, was significantly higher in KO group, indicating more robust tissue damage (Fig. 1H). These findings suggested that LTB4/BLT1 played an indispensable role in anti-influenza virus effects by attenuating inflammatory manifestations caused by the viral infection.

### The LTB4/BLT1 axis attenuates IL-1β production in macrophages exposed to influenza infection

**Fig. 2:**
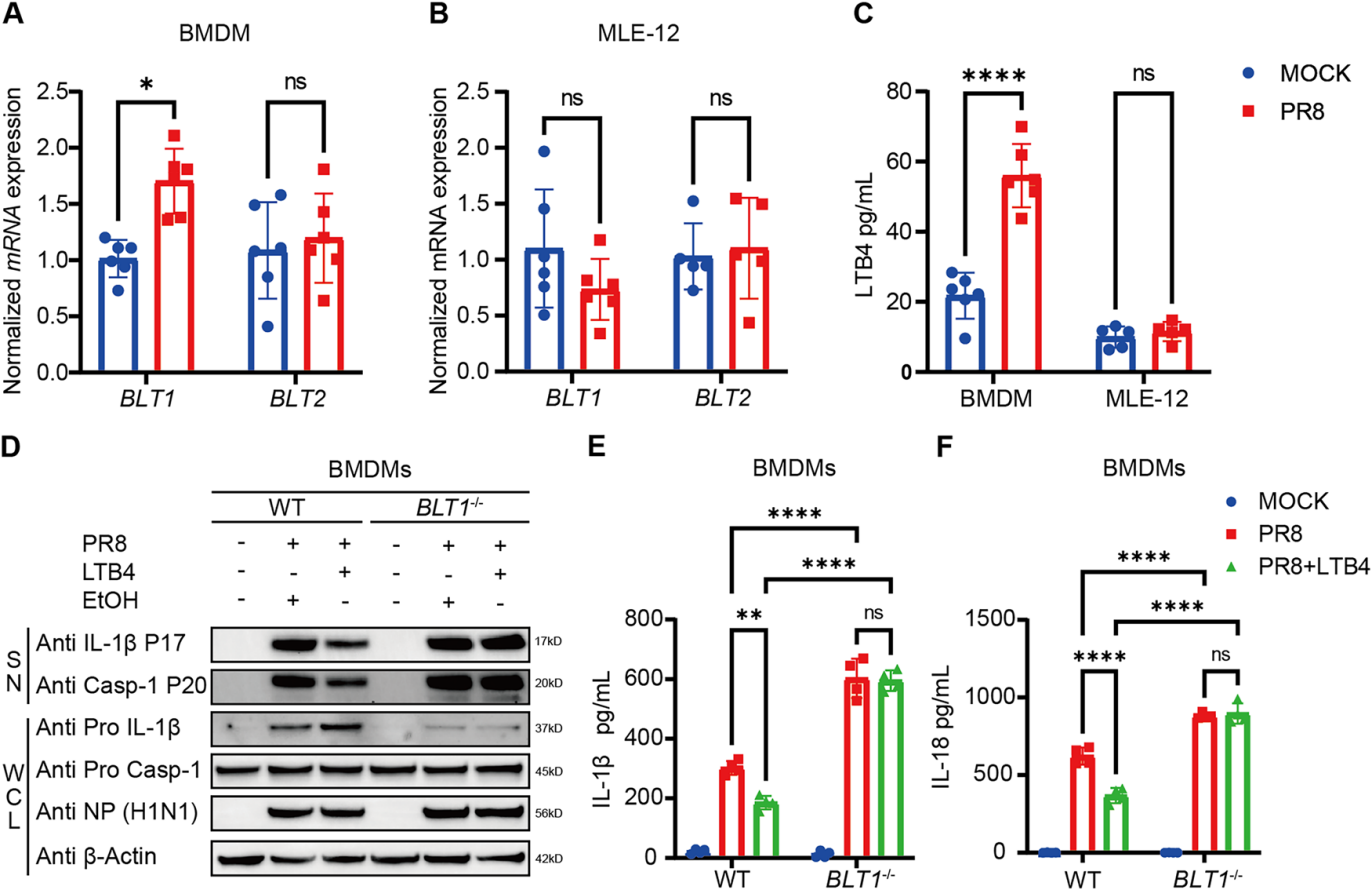
The LTB4-BLT1 axis attenuates IL-1β production in macrophages following influenza infection. **A** Expression levels of *BLT1* and *BLT2* were determined in BMDMs with or without IAV infection by reverse transcription qPCR at 16 h post-infection (*n* = 6 per group). (representative of three assays). **B** Expression levels of *BLT1* and *BLT2* were determined in MLE-12 with or without IAV infection by reverse transcription qPCR at 24 h post infection (*n* = 6 per group). (representative of three assays). **C** The LTB4 level in the BMDMs and MLE-12 cell culture supernatants was assessed by ELISA at 16 h (BMDMs) and 24 h (MLE-12) post infection (*n* = 6 per group). (representative of three assays). **D**–**F**, BMDMs isolated from WT and *BLT1*^*−/−*^ mice were primed with or without 100 nM LTB4 0.5 h, followed by infection with IAV (MOI = 20) for 16 h. **D** Cell supernatants and whole cell lysis were collected for immunoblot analysis. IL-1β (**E**) and IL-18 (**F**) release in the supernatants was determined by ELSIA, respectively. (representative of three assays). Statistics: mean ± SD; unpaired, two-tailed Student’s *t* test (**A**–**C)**; two-way ANOVA (**E**, **F**); *p < 0.05, **p < 0.01, ***p < 0.001 and ****p < 0.0001.

Following the determination of the heightened vulnerability of BLT1-deficient mice to IAV infection, we sought to identify the principal lung cell type implicated in this phenomenon. We re-analyzed single-cell RNA sequencing (scRNA-seq) data from GSE202325 to identify the cell types exhibiting *BLT1* expression in lung tissue from mice infected with IAV [29]. Our findings revealed that macrophages were the primary cell type with higher *BLT1* expression compared to others following infection (Fig. S1A). As expected, *BLT2* expression was difficult to detect (Fig. S1A). To further investigate this, we extracted bone marrow-derived macrophages (BMDMs) from wild-type mice. In addition, we also utilized MLE-12 cells as a model for murine alveolar epithelial cells. After IAV infection, we assessed the activation of the LTB4/BLT1 pathway at both transcriptional and expression levels (Fig. 2A–C). Notably, BMDMs demonstrated a substantial elevation in *BLT1* mRNA expression, whereas *BLT2* expression remained constant in both MLE-12 cells and BMDMs (Fig. 2A, B). Moreover, the content of LTB4 in cell supernatants was markedly elevated in BMDMs relative to MLE-12 cells (Fig. 2C), indicating that LTB4 primarily targets macrophages during influenza infection. Therefore, we concentrated our future investigations on macrophages. To further examine the involvement of LTB4 in IAV-induced macrophage inflammation, we infected WT and *BLT1*^−/−^ BMDMs with IAV. Following infection, *BLT1*^−/−^ BMDMs had a marked enhancement in inflammasome activation, indicated by increased caspase-1 cleavage and higher production of IL-1β and IL-18 into the cell culture supernatant (Fig. 2D–F). Furthermore, the supplementation of LTB4 in infected WT BMDMs, as compared to *BLT1*^−/−^ BMDMs, markedly decreased IL-1β and IL-18 concentrations in the supernatant and mitigated caspase-1 and IL-1β cleavage (Fig. 2D–F). Nonetheless, despite LTB4 treatment, *BLT2*^−/−^ iBMDMs (immortalized bone-marrow-derived-macrophages) demonstrated diminished inflammasome activation relative to WT BMDMs (Fig. S2A). These findings indicate that LTB4 restricts IAV-induced inflammasome activation through BLT1, but not BLT2, and that alveolar epithelial cells have a negligible role in this process.

### The LTB4/BLT1 axis mediates the NLRP3-dependent IL-1β release in IAV-infected BMDMs

**Fig. 3:**
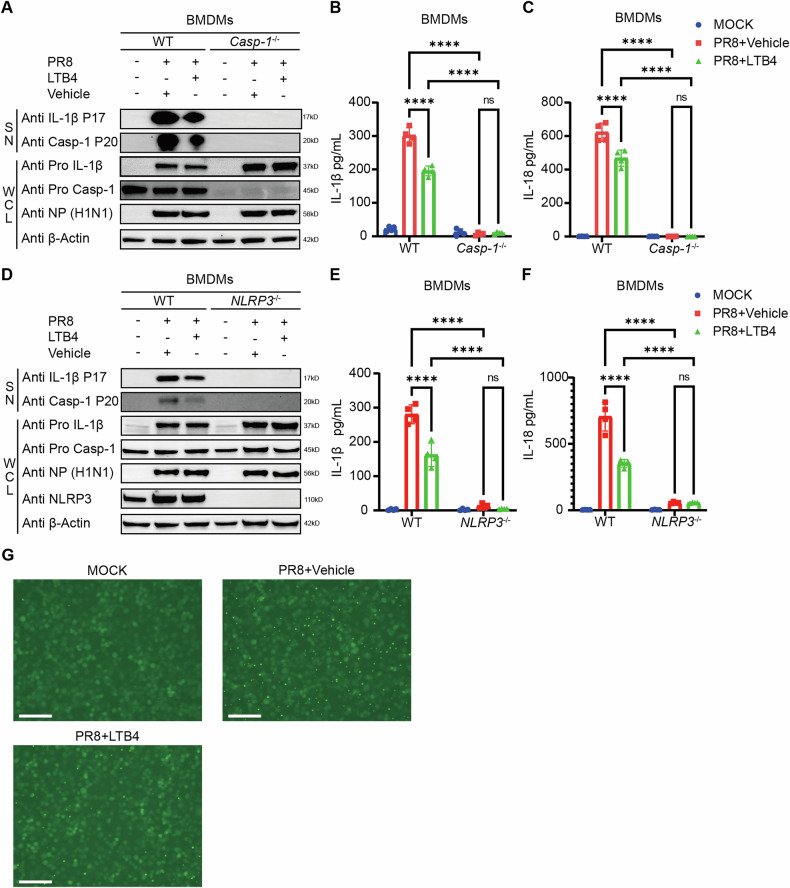
The LTB4/BLT1 axis mediates the NLRP3-dependent IL-1β release from BMDMs following influenza infection. **A**–**C**, BMDMs isolated from WT and *Caspase-1*^*−/−*^ mice were primed with or without 100 nM LTB4 0.5 h, followed by infection with IAV (MOI = 20) for 16 h. Cell supernatants and whole cell lysis were collected for immunoblot analysis (**A**). IL-1β (**B**) and IL-18 **(C)** release in the supernatants was measured by ELISA, respectively. (representative of three assays). **D**–**F**, BMDMs isolated from WT and *NLRP3*^*−/−*^ mice were primed with or without 100 nM LTB4 0.5 h, followed by infection with IAV (MOI = 20) for 16 h. Cell supernatants and whole cell lysis were collected for immunoblot analysis (**D**). IL-1β (**E**) and IL-18 (**F**) release in the supernatants was determined by ELSIA, respectively. (representative of three assays). **G** Representative immunofluorescence images of ASC speck formation in iBMDMs, expressing ASC-GFP, with or without 100 nM LTB4 primed, infected with IAV (MOI = 10) for 16 h. Scale bars represent 250 μm. (representative of two assays). Statistics: mean ± SD; two-way ANOVA (**B**, **C**, **E**, **F**); *p < 0.05, **p < 0.01, ***p < 0.001 and ****p < 0.0001.

Subsequently, we aimed to clarify the fundamental mechanism via which the LTB4/BLT1 axis modulates IL-1β secretion during IAV infection. The maturation of pro-IL-1β necessitates cleavage by caspase-1 or caspase-11 in macrophages [11]. Considering that caspase-11 has a negligible involvement in IAV-induced inflammasome activation, we concentrated on caspase-1 and analyzed inflammasome activation in WT and *caspase-1*^−/−^ BMDMs post-infection. Our findings revealed a substantial decrease in IL-1β and IL-18 synthesis in IAV-infected *caspase-1*^−/−^ BMDMs, accompanied by the lack of cleaved caspase-1 (Fig. 3A–C), suggesting that the LTB4/BLT1 axis operates through caspase-1. To investigate the role of LTB4 in inflammasome activation during IAV infection, we infected WT, *AIM2*−/−, and *NLRP3*^−/−^ BMDMs with IAV. The levels of cleaved caspase-1, together with the generation of IL-1β and IL-18, were similar in WT and *AIM2*^−/−^ BMDMs, irrespective of LTB4 supplementation, indicating that the LTB4/BLT1 axis does not influence AIM2 inflammasome activation (Fig. S3A). In contrast, infection of WT and *NLRP3*^−/−^ BMDMs demonstrated a marked reduction in caspase-1 cleavage and the secretion of IL-1β and IL-18 in *NLRP3*^−/−^ BMDMs (Fig. 3D–F). The findings demonstrate that IAV-induced inflammasome activation is dependent on NLRP3, and that the LTB4/BLT1 axis diminishes inflammasome activation in an NLRP3-dependent manner

The conclusion is additionally corroborated by the noted reduction in ASC-complex formation after LTB4 therapy during IAV infection (Fig. 3G). In conclusion, these findings indicate that the LTB4/BLT1 axis reduces IL-1β release in macrophages through a NLRP3/caspase-1-dependent mechanism during influenza infection.

### LTB4/BLT1 axis inhibits NLRP3 Inflammasome activation caused by IAV via the cAMP-PKA Pathway

**Fig. 4:**
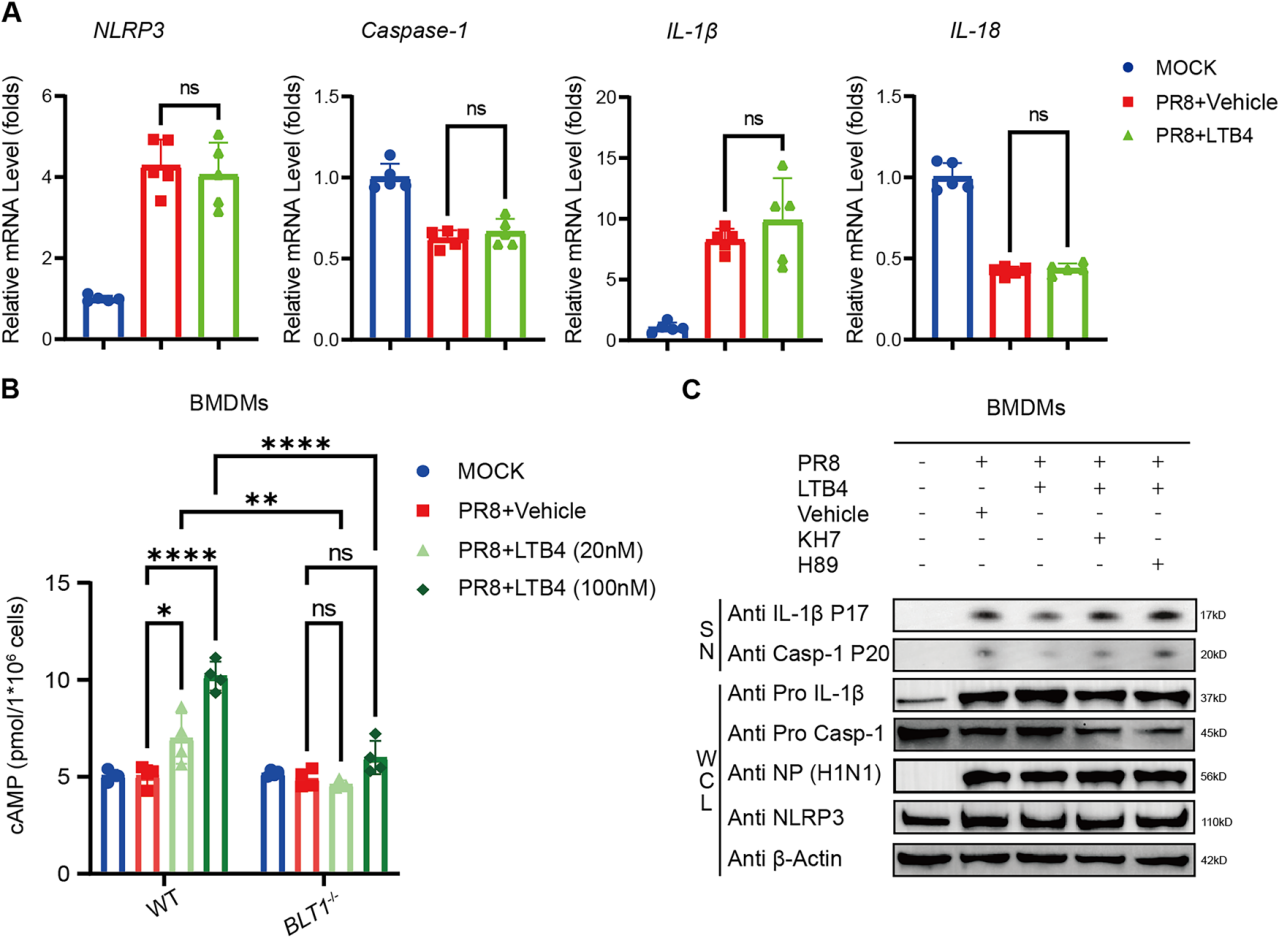
LTB4/BLT1 axis inhibits NLRP3 Inflammasome activation caused by IAV via the cAMP-PKA Pathway. **A** BMDMs isolated from WT mice were primed with or without 100 nM LTB4 0.5 h, followed by infection with IAV (MOI = 20) for 16 h. *NLRP3*, *Caspase-1*, IL-1β and *IL-18* expression in the infected cells were determined by qPCR (*n* = 5 per group). (representative of three assays). **B** cAMP induction in BMDMs isolated from WT and *BLT1*^*−/−*^ mice primed with or without 20 nM, 100 nM LTB4 0.5 h, followed by infection with IAV (MOI = 20) for 12 h. The concentration of cAMP in cells was quantified using ELISA. (representative of three assays). **C** BMDMs isolated from WT mice were primed with 100 nM LTB4, 5 μM KH7 or 5 μM H89 0.5 h, followed by infection with IAV (MOI = 20) for 16 h. Cell supernatants and whole cell lysis were collected for immunoblot analysis. (representative of three assays). Statistics: mean ± SD; unpaired, two-tailed Student’s *t* test (**A**); two-way ANOVA (**B**); *p < 0.05, **p < 0.01, ***p < 0.001 and ****p < 0.0001.

As demonstrated in our previous experiments, supplementation with LTB4 in WT BMDMs did not markedly influence the levels of pro-IL-1β or pro-caspase-1 (Fig. 3A, D). Furthermore, transcriptional analysis of genes linked to the NLRP3 inflammasome pathway demonstrated no notable alterations subsequent to LTB4 supplementation (Fig. 4A).

To examine the mechanism by which LTB4 diminishes NLRP3 inflammasome activation in IAV-infected BMDMs, we conducted RNA-seq analysis, revealing multiple changed pathways subsequent to LTB4 therapy (Fig. S4A). GSEA indicated a considerable increase and upregulate of the “cAMP-mediated pathway” in LTB4-treated BMDMs with IAV infection (Fig. S4B). Recently, multiple articles suggested that the LTB4/BLT1 axis could elevate intracellular cAMP level [30, 31]. Experimental evidence indicates that cAMP diminishes NLRP3 inflammasome activation by modulating the ubiquitination of NLRP3 [32], hence inhibiting NLRP3 inflammasome assembly. Consequently, we examined the function of the cAMP-PKA pathway in LTB4-mediated suppression of inflammasome activation during IAV infection. The LTB4 treatment significantly elevated cAMP levels in the infected WT BMDMs, but not in the infected *BLT1*^*−/−*^ BMDMs (Fig. 4B). In contrast, the increased cAMP levels in *BLT2*^*−/−*^ iBMDMs (Fig. S5A) were comparable to those in WT iBMDMs (Fig. 4B) following treatment with LTB4, thereby reinforcing the insignificance of BLT2 in the reduced NLRP3 inflammasome activation induced by LTB4. Previous studies have shown that elevated cAMP synthesis can inhibit caspase-1 cleavage and the generation of IL-1β and IL-18 through the cAMP/PKA pathway [32]. Consequently, we proposed that the cAMP/PKA pathway may facilitate the inhibitory influence of LTB4 on NLRP3 inflammasome activation. Our findings indicated that the adenylate cyclase (AC) inhibitor KH7 and the selective PKA inhibitor H89 were capable of obstructing the LTB4-induced the inhibition of NLRP3 inflammasome activation (Fig. 4C). The findings indicated that the LTB4/BLT1 axis suppressed NLRP3 inflammasome activation through a cAMP/PKA-dependent mechanism during IAV infection.

### LTB4/BLT1 axis promotes NLRP3 ubiquitination post-IAV infection

**Fig. 5:**
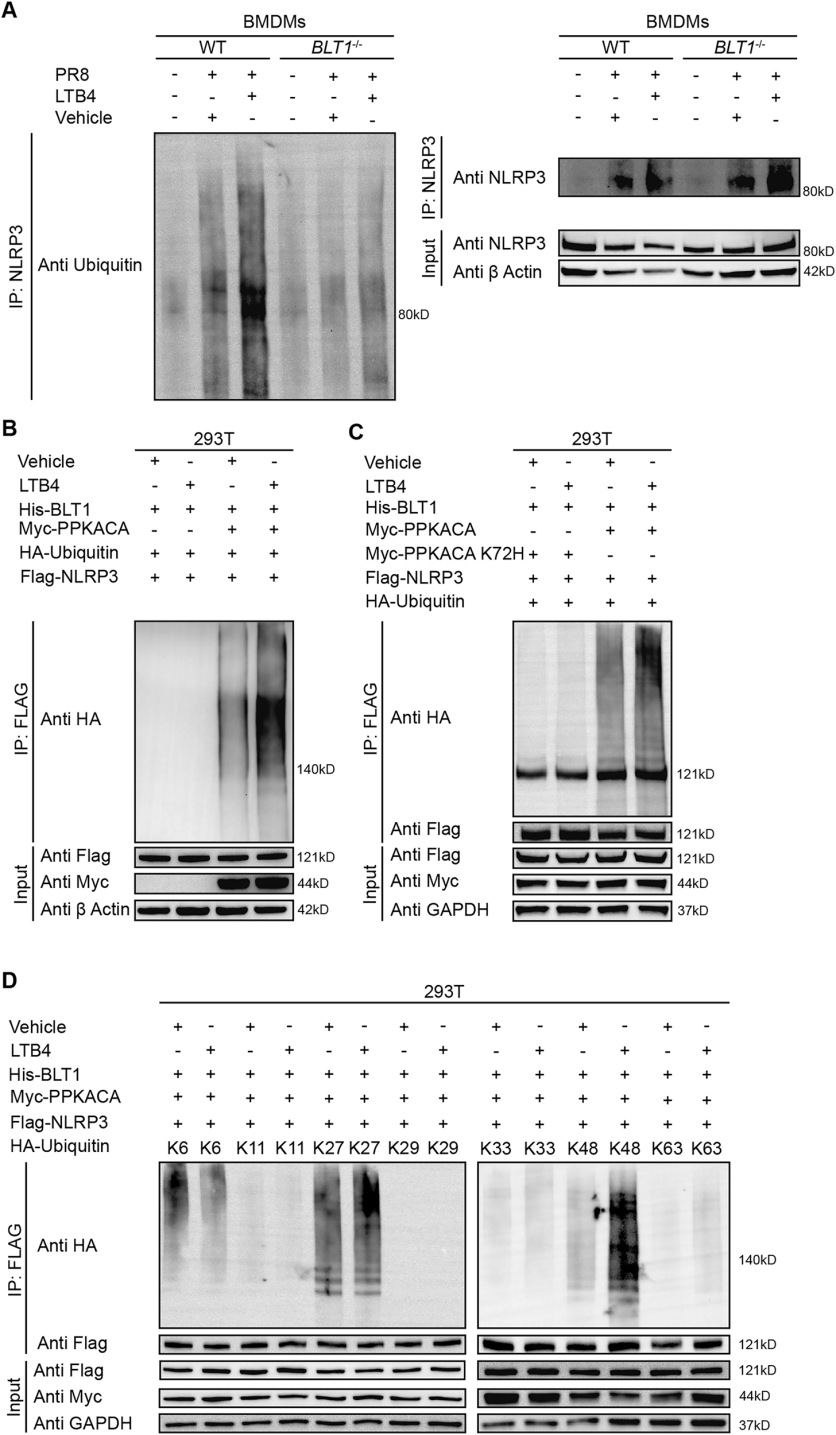
LTB4/BLT1 axis promotes NLRP3 ubiquitination following IAV infection. **A** BMDMs isolated from WT and *BLT1*^*−/−*^ mice were primed with 100 nM LTB4 0.5 h, followed by infection with IAV (MOI = 20) for 16 h. NLRP3 immunoprecipitants were analyzed for ubiquitination. (representative of three assays). **B** HEK293T cells expressing His-BLT1, Flag-NLRP3, HA-Ubiquitin (HA-Ub) and Myc-PPKACA or not, were treated with 100 nM LTB4 as indicated for 30 min. Flag-NLRP3 immunoprecipitants were analyzed for ubiquitination. (representative of three assays). **C** HEK293T cells expressing His-BLT1, Flag-NLRP3, HA-Ub, and Myc-PPKACA or Myc-PPKACA K72H were treated with 100 nM LTB4 for 30 min. Flag-NLRP3 was immunoprecipitated with anti-Flag for ubiquitination. (representative of three assays). **D** Immunoblots of the ubiquitinated NLRP3 in HEK293T cells transfected with His-BLT1, Flag-NLRP3, and Myc-PPKACA along with HA-Ub or its mutants. (representative of three assays).

Recent investigations have shown that ubiquitination is a crucial regulatory mechanism in inhibiting NLRP3 inflammasome activation [32–35]. The cAMP has been demonstrated to promote NLRP3 ubiquitination through PKA, thereby hindering its activation [32]. However, the interaction among the LTB4/BLT1 axis, the cAMP/PKA pathway, and NLRP3 ubiquitination has yet to be clarified. Our results demonstrate that LTB4 treatment promotes NLRP3 ubiquitination in IAV-infected WT BMDMs, whereas this effect is absent in infected *BLT1*^*−/−*^ BMDMs (Fig. 5A). To further examine this phenomenon, we conducted immunoprecipitation of NLRP3 under denaturing conditions using whole-cell lysates from WT and *BLT1*^*−/−*^ BMDMs. High-molecular-weight NLRP3 were detected following LTB4 supplementation in WT BMDMs, which were not present in *BLT1*^*−/−*^ BMDMs. The results indicate that NLRP3 experiences post-translational changes, including polyubiquitination, in a BLT1-dependent manner (Fig. 5A).

In accordance with our hypothesis, LTB4 increased NLRP3 ubiquitination in HEK293T cells co-expressing Flag-NLRP3, HA-ubiquitin, His-BLT1, and Myc-PRKACA (the catalytic subunit α of cAMP-dependent protein kinase). Significantly, NLRP3 ubiquitination was inhibited in cells devoid of Myc-PRKACA expression (Fig. 5B). Furthermore, the expression of the kinase-dead PRKACA-K72H mutant in HEK293T cells entirely obstructed LTB4-induced NLRP3 polyubiquitination (Fig. 5C), underscoring the critical importance of PKA kinase activity in this mechanism.

Considering that distinct polyubiquitin linkages yield specific functional outcomes, we further investigated the characteristics of NLRP3 ubiquitination. HEK293T cells were transfected with Flag-NLRP3, Myc-PRKACA, and His-BLT1, in conjunction with various HA-ubiquitin mutants (K6, K11, K27, K29, K33, K48, and K63), each allowing only one lysine residue to act as a ubiquitination site. K6-, K27-, K33-, K48-, and K63-linked ubiquitination were detected on NLRP3, while LTB4 specifically enhanced K27- and K48-linked polyubiquitination of NLRP3 (Fig. 5D). The data collectively indicate that the LTB4/BLT1 axis positively influences K27- and K48-linked polyubiquitination of NLRP3 through the cAMP/PKA pathway, thereby hinder its activation.

### The LTB4/BLT1 axis suppresses NLRP3 inflammasome activation by ubiquitinating K886 and K1023 sites on NLRP3

**Fig. 6:**
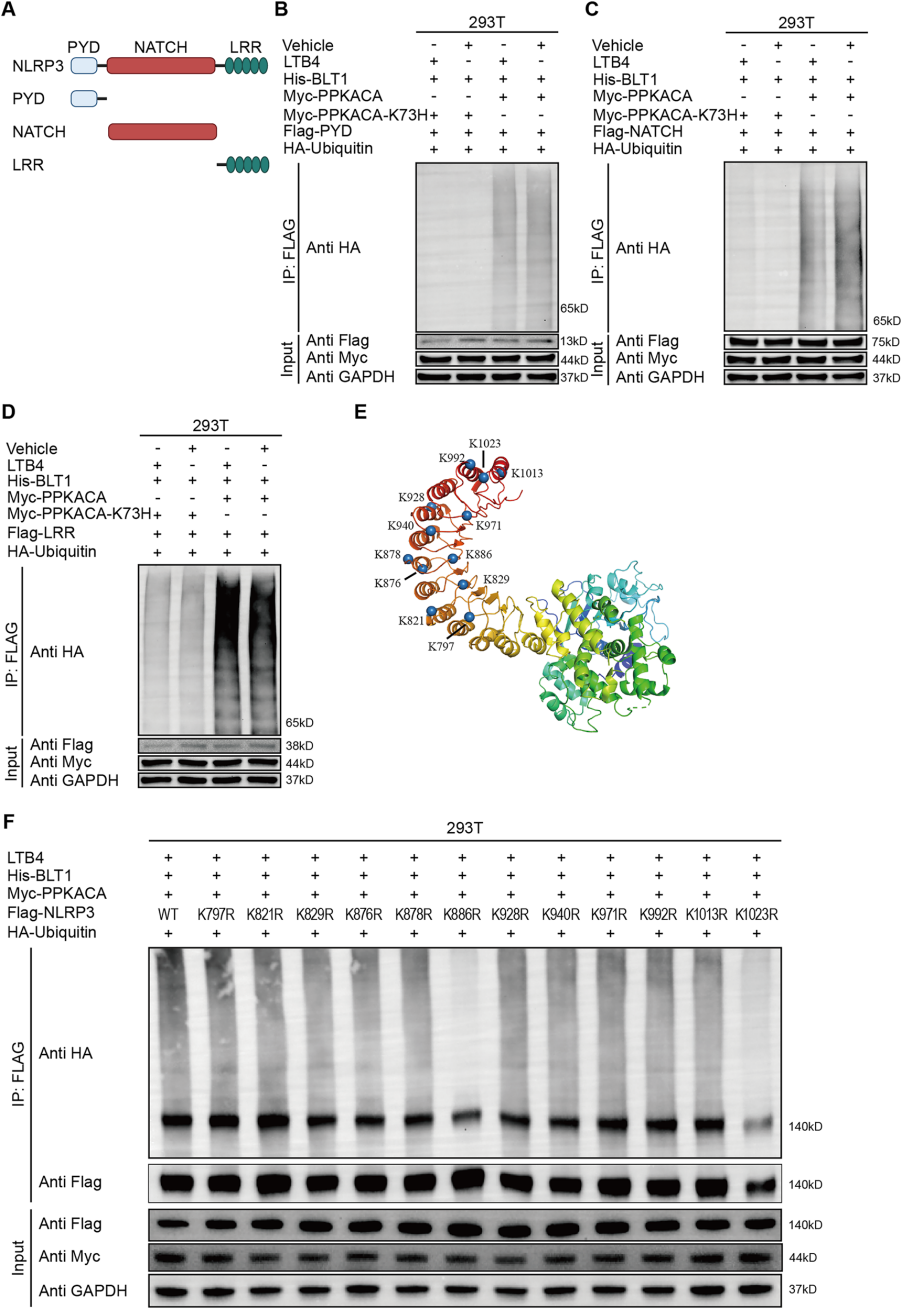
The LTB4/BLT1 axis suppresses the activation of NLRP3 inflammasome by ubiquitinating K886 and K1023 sites on NLRP3 through the cAMP/PKA signaling pathway. **A** Schematic diagram of NLRP3 and its truncation mutants. **B**–**D**, HEK293T cells expressing His-BLT1, Myc-PRKACA, HA-Ub, and Flag-NLRP3 PYD (**B**), NACHT (**C**), or LRR (**D**) were immunoprecipitated with anti-Flag for ubiquitination (representative of three assays). **E**, **F**, Immunoblots of the ubiquitinated NLRP3 in HEK293T cells transfected with His-BLT1, Myc-PPKACA and HA-Ub along with Flag-NLRP3 or its mutants. (representative of three assays).

Recent studies have demonstrated that NLRP3 ubiquitination takes place at various NLRP3 lysine residues, hence modulating NLRP3 inflammasome activation [32, 36]. To identify the precise domain undergoing ubiquitination, we created Flag-tagged constructs for the pyrin domain (PYD), nucleotide-binding domain (NATCH), and leucine-rich repeat (LRR) domain of NLRP3 (Fig. 6A). Upon expressing these constructs in HEK293T cells, we observed that LTB4-induced ubiquitination of NLRP3 through the cAMP/PKA route primarily transpired within the LRR domain (Fig. 6B–D). We subsequently aimed to pinpoint the precise lysine residues inside the LRR domain that facilitate ubiquitination. A series of NLRP3 mutants was produced, wherein lysine residues within the LRR domain (Lys797, Lys821, Lys829, Lys876, Lys878, Lys886, Lys928, Lys940, Lys971, Lys992, Lys1013 and Lys1023) of NLRP3 were replaced with arginine, resulting in 12 single-site mutants (K797R, K821R, K829R, K876R, K878R, K886R, K928R, K940R, K971R, K992R, K1013R and K1023R) (Fig. 6E). Of these mutants, only K1023R shown somewhat reduced expression (Fig. 6F). The evaluation of NLRP3 ubiquitination in HEK293T cells demonstrated that the K886R and K1023R mutations markedly inhibited ubiquitinationNLRP3, while the other NLRP3 eight mutants exhibited no discernible impact (Fig. 6F). The results demonstrate that Lys886 and Lys1023 are essential residues NLRP3necessary for the K27- and K48-linked ubiquitination of NLRP3 facilitated by the LTB4/BLT1 pathway.

### Exogenous LTB4 treatment diminishes NLRP3 inflammasome activation and enhances survival after IAV infection

**Fig. 7:**
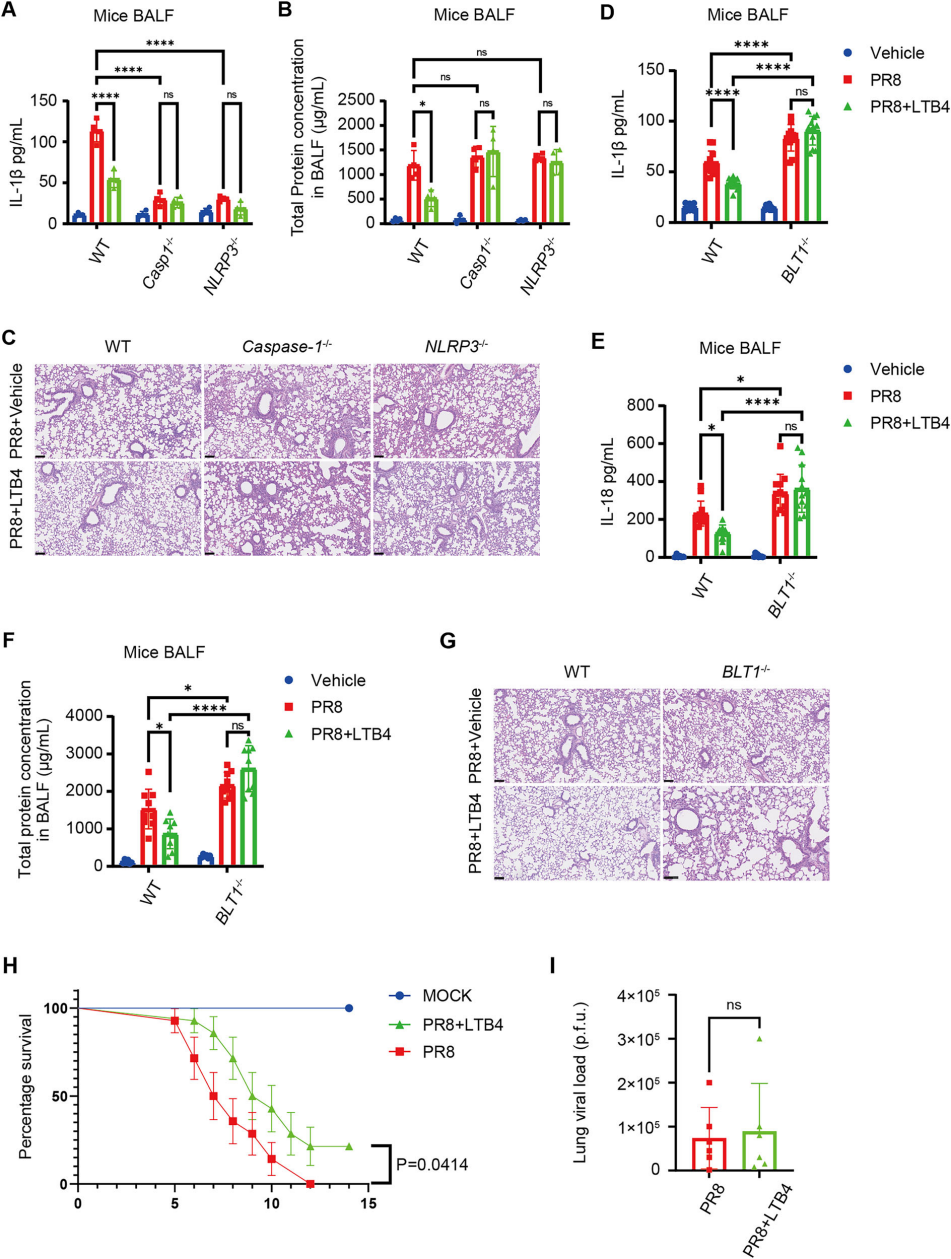
The exogenous treatment of LTB4 suppresses NLRP3 inflammasome activation and improves survival after IAV infection. **A**–**C** WT, *Caspase-1*^*−/−*^ and *NLRP3*−/− mice were infected with IAV (5,000 pfu) with or not a treatment of 300 ng LTB4 at 4 days post infection, and the level of IL-1β (**A**) and total protein levels (**B**) in the BALF were assessed by ELISA at 5 days post-infection. Micrographs of haematoxylin and eosin-stained lung sections (**C**) prepared at 5 days after IAV infection. Scale bars represent 100 µm. (uninfected, n = 6; IAV, n = 12 mice per group) (representative of three assays). WT and *BLT1*^*−/−*^ mice were infected with IAV (5000 pfu) with or not a treatment of 300 ng LTB4, and the level of IL-1β (**D**), IL-18 (**E**) and total protein levels (**F**) in the BALF were measured using ELISA at 5 days post-infection. Micrographs of haematoxylin and eosin-stained lung sections (**G**) prepared at 5 days after IAV infection. Scale bars represent 100 µm. (uninfected, *n* = 6 or 9; IAV, *n* = 12; IAV plus LTB4 treatment, *n* = 12 mice per group) (representative of three assays). **H** WT mice were infected with an LD_100_ dose (15,000 pfu) of IAV with a treatment of 300 ng LTB4 or vehicle at 4 days post-infection, and survival was monitored (uninfected, *n* = 4; IAV, *n* = 14 mice per group). (representative of three assays). **I** WT mice were infected with an LD_100_ dose (15,000 pfu) of IAV with a treatment of 300 ng LTB4 or vehicle at 4 days post-infection, the lungs were harvested for pulmonary viral titers analysis (n = 6 mice per respective group). (representative of three assays). Statistics: mean ± SD; two-way ANOVA (**A**, **B**, **D**, **E**, **F**), unpaired, or two-sided log-rank test (**H**); *p < 0.05, **p < 0.01, ***p < 0.001 and ****p < 0.0001.

Considering that LTB4 treatment significantly reduced NLRP3 inflammasome activity in IAV-infected macrophages, we then examined whether LTB4 administration could provide in vivo protection against deadly IAV infection. To simulate delayed therapeutic intervention, we gave LTB4 intranasally (300 ng LTB4) four days post-infection. In accordance with our in vitro results, LTB4 therapy markedly decreased IL-1β levels in the BALF of infected WT mice, but did not affect *Caspase-1*^*−/−*^, *NLRP3*^*−/−*^, or vehicle-treated mice (Fig. 7A). Likewise, total protein content in bronchoalveolar lavage fluid and lung pathology scores demonstrated decreased pulmonary damage after to LTB4 treatment (Fig. 7B, C). Nonetheless, LTB4 therapy did not safeguard *BLT1*^*−/−*^ mice from IAV infection, as indicated by similar levels of IL-1β (Fig. 7D) and IL-18 (Fig. 7E), total protein concentration in BALF (Fig. 7F), and lung histological damage (Fig. 7G) between the treated and untreated groups. Moreover, LTB4 therapy markedly enhanced survival rates in IAV-infected WT mice, while exhibiting no protective effect in *BLT1*^*−/−*^ mice (Fig. 7H). Significantly, the LTB4 treatment did not modify viral burden in the lungs, as viral titers were consistent across treated and untreated WT mice (Fig. 7I). These data emphasize that the protective effects of LTB4 are predominantly facilitated by its immunoregulatory actions rather than direct antiviral activity. Our findings collectively indicate that targeting the LTB4/BLT1 axis efficiently reduces influenza virus-induced mortality by inhibiting NLRP3 inflammasome activation in vivo.

## Discussion

Influenza pneumonia has historically resulted in numerous fatalities and is a considerable worldwide health risk. Antiviral therapy alone frequently fails to alleviate the pronounced inflammatory response triggered by IAV infection, especially in severe cases [2, 3]. This study discovered LTB4 as a novel immunoregulatory molecule that reduces proinflammatory cytokine release, mitigates pulmonary pathological injury, and enhances survival outcomes in a mouse model of deadly IAV infection. Our data indicate that LTB4 confers protective effects via diminishing NLRP3 inflammasome activation through the BLT1-cAMP-PKA pathway, consequently inhibiting the release of downstream cytokines IL-1β and IL-18. Evidence indicates that the suppression of the NLRP3 inflammasome is predominantly facilitated by ubiquitinationNLRP3, rather than by other post-translational changes such phosphorylation, acetylation, or SUMOylation [34, 35, 37]. Moreover, our study of NLRP3 demonstrated that the LRR domain is essential for LTB4-mediated inhibition of NLRP3 inflammasome activation. Utilizing targeted lysine mutagenesis, we identified K886 and K1023 as critical locations for K27- and K48-linked polyubiquitination, a process catalyzed by increased intracellular cAMP levels.

Previous research reveals that LTB4 augments IFN-α production in the interstitial macrophages of IAV-infected mice via BLT1 signaling, thus inhibiting the proliferation of monocyte-derived macrophages and enhancing host tolerance to virus-induced inflammation [24]. Our research further substantiates the protective function of LTB4 against IAV infection through a unique pathway. LTB4 does not directly alter virus titers in infected lungs, as evidenced by the unchanged viral load in treated mice. Instead, its protective effects are achieved by modulating the host immune response, specifically by dampening virus-induced inflammation. LTB4 reduces the excessive release of inflammatory cytokines from infected pulmonary tissues, which is accompanied by decreased diffuse alveolar damage, ultimately preserving lung function and improving survival in infected mice. These findings highlight its potential as a therapeutic strategy to control immunopathology during severe influenza infection. Although the BLT1 is extensively expressed in mammalian cells, our findings indicate that macrophages, rather than epithelial cells, serve as the principal mediators of this regulatory mechanism. Furthermore, our results underscore the essential function of BLT1, as opposed to BLT2, in facilitating the protective effects of LTB4 during IAV infection. A key strength of our investigation is the clarification of the unique molecular pathway through which LTB4 regulates inflammasome activation, the particular NLRP3 post-translational changes implicated, and the precise lysine residues that are ubiquitinated. To validate our findings, we performed extensively in vitro and in vivo investigations. After confirming the diminished NLRP3 inflammasome activation and proinflammatory cytokine release by LTB4 in BMDMs, we utilized *NLRP3*^*−/−*^*, Caspase-1*^*−/−*^, and *BLT1*^*−/−*^ mice to validate the essentiality of these pathways in LTB4-mediated protection against IAV infection. Although earlier studies have mainly concentrated on the phosphorylation of NLRP3 as a crucial regulatory mechanism [32, 33, 38], our research highlights the essential function of site-specific ubiquitination in regulating NLRP3 activation during LTB4 therapy. LTB4 has lately emerged as a prospective therapeutic target, with current phase II clinical trials evaluating its efficacy [39]. Growing evidence indicates that heightened LTB4 levels provide resistance to many infections [23, 24, 40]. Utilizing a mouse model of IAV infection, we found that LTB4 mitigates PR8-induced tissue damage. However, our research exclusively focused on the PR8 strain, necessitating additional confirmation across various influenza strains and subtypes. Moreover, several studies indicate that LTB4 exerts an immunoregulatory function in many immune cell types, including neutrophils and T cells, in scenarios such as cystic fibrosis and fungal infections [27, 41, 42]. Further research is required to explore the potential role of LTB4 in various immune cell types after IAV infection.

In conclusion, our study confirms the inhibitory function of LTB4 in regulating excessive inflammatory responses during influenza virus infection. We recognize LTB4 as a principal regulator of NLRP3 inflammasome activation via the BLT1 (LTB4 receptor 1), with subsequent activation of the cAMP/PKA pathway promoting K27- and K48-linked polyubiquitination of NLRP3 at K886 and K1023. These findings offer novel insights into the immunomodulatory roles of LTB4 and underscore its potential as a therapeutic target for alleviating inflammatory tissue damage during viral infections.

## Methods

### Antibodies and reagents

The anti-GAPDH (AC054) antibody and anti-β-Actin (AC028) antibody were obtained from ABclonal Technology. The anti-Caspase-1 P20 (AG-20B-0042) antibody and anti-NLRP3 (AG-20B-0006) antibody were purchased from Adipogen Life Science. The anti-NP (H1N1) (GTX125989) antibody was obtained from Genetex. Antibodies for FLAG (M185-7), MYC (M192-7), HA (M180-7) were all purchased from MBL. The anti-IL-1β (M185-7) antibody was obtained from Proteintech. The anti-Ubiquitin (sc-8017) antibody was purchased from Santa Cruz Biotechnology. LTB4 (20110) and KH7 (13243) were obtained from Cayman Chemical. Ethanol (E130059) was purchased from Aladdin. H89 (HY-15979) was obtained from MCE.

### Viruses

All in vitro and in vivo infection were performed using Influenza A/Puerto Rico/8/34 (PR8), kindly provided by Prof. Jing-Ren Zhang (Tsinghua University, China). The PR8 virus was propagated in d in 9-day-old specific pathogen-free (SPF) chicken embryos and titrated on MDCK cells using standard plaque assays.

### Mice and animal models

The C57BL/6J^Gpt-Ltb4r1em1Cd2118/Gpt^ (*Lrb4r1*^*−/−*^ mice; Strain NO. T006466) and C57BL/6J^Gpt-Nlrp3em7Cd4411/Gpt^ (*NLRP3*^*−/−*^ mice; Strain NO. T010873) were purchased from Gempharmatech (Jiangsu, China). The *Caspase-1*^*−/−*^ and *AIM2*^*−/−*^ mice were kindly provided by Prof. Feng Shao (National Institute of Biological Sciences, China). Mice were housed at 18–23 °C with 40–60% humidity under a normal 12 h light–dark cycle with food and water available ad libitum, in the specific-pathogen-free facility at Institute of Biophysics of Chinese Academy of Sciences (Beijing, China). All animal experiments were carried out in accordance with the Statute on the Administration of Laboratory Animals by the Ministry of Science and Technology of China, using protocols approved by the institutional Animal Care and Use Committee at the Institute of Biophysics of Chinese Academy of Sciences (approval number SYXK2023190 and ABSL-2-2023026). Experimental animals were randomly allocated to treatment groups using a computer-generated randomization sequence (random number table method) to ensure unbiased group assignment. All experimental animals were included in accordance with pre-defined protocols, and no subjects were excluded during the study.

For in vivo infection, 6–8-week-old male C57/B6 mice were challenged intranasally (in 30 μL DPBS) with IAV at a LD50 dose of 5000 pfu or a LD100 dose of 15,000 pfu, respectively.

### Cell culture, differentiation and Infection

Mouse bone marrow-derived macrophages (BMDMs) were generated according to the previously described method [43]. iBMDM-ASC-GFP cells (immortalized bone marrow-derived macrophages) were kindly provided by Prof. Di Wang (Zhejiang University, China). Human embryonic kidney (HEK) 293T cells (Procell, China) were cultured in Dulbecco’s modified Eagle’s medium (DMEM; Gibco, USA). MDCK cells (Procell, China) and L929 cells (Procell, China) were cultured in minimum essential medium Eagle (MEM; Gibco, USA). Cells were routinely cultured at 37 °C with 5% CO_2_, and all media were supplemented with 10% fetal bovine serum (FBS; Gibco, USA). All cell lines used in this study were routinely tested for Mycoplasma contamination and only Mycoplasma-free cells were used in the experiments.

For in vitro infection, cells were seeded in tissue culture plates the day before infection and randomly allocated to experimental groups using a computer-generated randomization table. Subsequently, cells were inoculated with IAV at a 20 multiplicity of infection (MOI) in fresh and serum-free DMEM for virus infection. After adsorption for 1.5 h at 37 °C, the cells were washed with Dulbecco’s phosphate-buffered saline (D-PBS; Gibco, USA) and cultured in DMEM containing 10% FBS.

### Plasmid construction and transfection

Plasmids encoding Ubiquitin including Ub-K6, Ub-K11, Ub-K27, Ub-K29, Ub-K33, Ub-K48, Ub-K63, NLRP3 including NLRP3-PYD, NLRP3-NATCH, NLRP3-LRR, PPKACA including the kinase-dead PRKACA-K72H mutant were kindly provided by Prof. Di Wang (Zhejiang University, China). Truncated mutant vectors NLRP3 (K797R, K821R, K829R, K876R, K878R, K886R, K928R, K940R, K971R, K992R, K1013R and K1023R) were constructed using the Mut Express II Fast Mutagenesis Kit V2 (C214-01, Vazyme, China) according to the manufacturer’s instructions. Plasmids encoding all plasmid DNAs for transfection were prepared using QIAGEN Plasmid Plus Midi Kit (12945, QIAGEN, Germany). HEK293T cells were transiently transfected with Lipofectamine 3000 (L3000015, Invitrogen, USA) according to the manufacturer’s instructions.

### Lung tissue histology and injury scoring

The lung samples were promptly fixed in a 4% formalin solution, subsequently embedded in paraffin, and then sectioned into slices with a thickness of 4 μm for the purpose of histopathologic analysis. These sections were stained using hematoxylin and eosin (H&E) to facilitate examination under light microscopy. The slides were randomized, read in a blinded manner, and subsequently assessed using a semi-quantitative scoring system as previously described [4]. Edema, alveolar and interstitial inflammation, alveolar and interstitial hemorrhage, atelectasis, necrosis, and hyaline membrane formation were each assigned scores on a scale ranging from 0 to 4: absence of injury received a score of 0; presence of injury in 25% of the field received a score of 1; presence of injury in 50% of the field received a score of 2; presence of injury in 75% of the field received a score of 3; and presence of injury throughout the entire field received a score of 4. The results were validated by an experienced and qualified pathologist.

### RNA isolation and reverse transcription quantitative PCR (qPCR)

Total RNA from the mouse lungs was extracted using TRIzol Reagent (Invitrogen; USA) and was treated with DNase I (Invitrogen; USA) according to the instructions provided by the manufacturer. Total RNA from BMDMs was extracted by an RNA isolation kit (Vazyme; China). First-strand complementary DNA was synthesized from 200 ng of total RNA using a RevertAid First Strand cDNA Synthesis Kit (Thermo Scientific; USA). Generated cDNA was subjected to qPCR in a 20 μL reaction volume using a PowerUp SYBR Green Master Mix (Applied Biosystems; USA). Cq values obtained on a CFX96 PCR System (Bio-Rad) were analyzed using the 2^−ΔΔCq^ formula by normalizing the target gene expression to *GAPDH* or *β-Actin*.

The following primers were used:

*β-Actin* For 5′ AACAGTCCGCCTAGAAGCAC 3′

*β-Actin* Rev 5′ CGTTGACATCCGTAAAGACC 3′

*GAPDH* For 5′ AGGTCGGTGTGAACGGATTTG 3′

*GAPDH* Rev 5′ TGTAGACCATGTAGTTGAGGTCA 3′

*NLRP3* For 5′ ATTACCCGCCCGAGAAAGG 3′

*NLRP3* Rev 5′ TCGCAGCAAAGATCCACACAG 3′

*IL-1β* For 5′ TGGACCTTCCAGGATGAGGACA 3′

*IL-1β* Rev 5′ GTTCATCTCGGAGCCTGTAGTG 3′

*IL-18* For 5′ GACTCTTGCGTCAACTTCAAGG 3′

*IL-18* Rev 5′ CAGGCTGTCTTTTGTCAACGA 3′

*Caspase-1* For 5′ CACAGCTCTGGAGATGGTGA 3′

*Caspase-1* Rev 5′ TCTTTCAAGCTTGGGCACTT 3′

*BLT1* For 5′ ATGGCTGCAAACACTACATCTCCT 3′

*BLT1* Rev 5′ CACTGGCATACATGCTTATTCCAC 3′

*BLT2* For 5′ ACAGCCTTGGCTTTCTTCAG 3′

*BLT2* Rev 5′ TGCCCCAATTACTTTCAGCTT 3′

### Immunoblotting and Immunoprecipitation

For Immunoblotting, cells were lysed in RIPA buffer (89901, Thermo Scientific, USA) with 1× Halt Protease and Phosphatase Inhibitor Cocktail (78440, Thermo Scientific, USA) and incubated on a rocker with ice for 30 min and then centrifuged at 12,000 × g, 4°C for 10 min to obtain cell lysates. Cell lysates were boiled at 70 °C for 10 min in 4× LDS loading buffer and resolved by NuPAGE Bis-Tris Mini Gels (Invitrogen, USA).

For immunoprecipitation, whole-cell lysates were lysed by Pierce™ IP buffer (87787, Thermo Scientific, USA) with Protease and Phosphatase Inhibitor (MCE, USA) and then incubated with Anti-DDDDK-tag mAb-Magnetic Agarose beads (M185-10, MBL, Japan) or appropriate antibodies plus Protein A/G beads (Pierce). The beads were washed three to five times with low-salt lysis buffer, and then the immunoprecipitants were resuspended in 30 μL 2× LDS loading buffer and boiled at 70 °C for 10 min and resolved by NuPAGE Bis-Tris Mini Gels.

### Enzyme-linked immunosorbent assay (ELISA)

BALF was collected by cannulating the trachea with a 22-gauge needle and washing the lung with 1 mL of cold, sterile DPBS, and then centrifuged (1500 × *g*; 10 min) at 4 °C. The cell culture supernatants were harvested and centrifuged at 4 °C, 1500 × *g* for 10 min. For cell lysate, cells were washed by DPBS twice and lysed by Cell Lysis Buffer II (Invitrogen; USA) and centrifuged. Then supernatants were collected and precipitations were discarded. IL-1β, IL-18, LTB4 and cAMP levels were measured by an IL-1β Mouse Uncoated ELISA Kit (Invitrogen; USA), IL-18 Mouse Uncoated ELISA Kit (Invitrogen; USA), LTB4 ELISA and cAMP ELISA kit (Enzo Life Sciences; USA), respectively. LTB4 and cAMP levels were assessed by ELISA (Enzo Life Sciences; USA). The total protein content was assessed by Pierce Rapid Gold BCA Protein Assay Kit (Thermo Fisher Scientific; USA).

### Transcriptomic analysis

The analysis was conducted in R using the Seurat package (v5.0). Quality control was applied to filter cells based on the following criteria: cells with more than 200 and fewer than 3500 detected genes, and less than 10% mitochondrial gene content were retained. SCTransform and canonical correlation analysis (CCA) were then used. Clustering was carried out by Seurat FindClusters function with a resolution of 0.2. Cell type annotation was subsequently performed using the SingleR package.

For bulk RNA-seq, BMDMs from two mice were treated as previously described and then pooled. Total RNA in BMDMs infected by PR8 with or without LTB4 was extracted via a standardized Trizol method, and then quantified and qualified. mRNA libraries were constructed by TruSeq RNA Library Prep Kit (Illumina, USA) and then sequenced by Illumina NovaSeq 6000 platform. Raw data were filtered using Fastp v2.1.0 and then mapped to mouse genome GRCm39. Gene expression counts were finally obtained by featureCounts v1.5.0-p2. Subsequent analyses were performed in R v4.3.0. EdgeR v4.0.1 was used for identifying differentially expressed genes (DEGs) with |foldchange| > 2. KEGG enrichment analysis and gene set enrichment analysis (GSEA) was performed using clusterProfiler v4.10.0 and visualized by ggplot2 v 4.10.0.

### Statistical analysis

Parametric tests (Student’s t-test and ANOVA) were selected after confirming normality with Shapiro-Wilk tests (*p* > 0.05 for all datasets), with variance homogeneity verified using Levene’s test prior to ANOVA. When assumptions were violated, non-parametric alternatives were applied: Mann-Whitney U test for two-group comparisons and Kruskal-Wallis test with Dunn’s post-hoc analysis for multiple comparisons. For instances where variances differed significantly (F > 2.5 by F-test), Welch’s ANOVA with adjusted degrees of freedom was employed.

All experiments were independently repeated at least two or three times with complete inclusion of experimental animals and data points. Investigators remained blinded to group allocation during both data collection and outcome assessment, while animal grouping and treatment administration were performed by separate personnel.

The survival curves were generated using the Kaplan–Meier method, and statistical analyses were conducted employing the log-rank test. Student’s t-tests were utilized to assess the statistical significance between two groups. For comparisons involving three or more groups, analyses were performed using one-way or two-way ANOVA. The statistical analysis method of each experiment was reported in the figure legends. A two-sided *p* value of less than 0.05 was considered statistically significant. All the statistics were performed using the software GraphPad Prism v9.5.

## Supplementary information

Supplementary figure legends

Figure S1

Figure S2

Figure S3

Figure S4

Figure S5

Author Contribution Statement

## Data Availability

The raw RNA-seq data presented in this paper have been deposited in the National Genomics Data Center under the accession number CRA022967.

## Code availability

All computational codes can be accessed under https://github.com/PCCM-CaoLab/LTB4-NLRP3-and-influenza .
