# Supplementary material for: The LTB4-BLT1 axis attenuates influenza-induced lung inflammation by suppressing NLRP3 activation
Source: Cell Death Discov. 2025 Apr 6;11:148. doi: 10.1038/s41420-025-02450-8 (PMC11973165; doi:10.1038/s41420-025-02450-8)
Supplement: Supplementary file 1 — Supplementary figure legends [file 41420_2025_2450_MOESM1_ESM.docx]

**Fig. S1 | scRNA-seq analysis of IAV infected mouse (GSE202325)**

**A,** UMAP plot of a single-cell RNA sequencing (scRNA-seq) dataset from mouse lung tissue, 3 days post-infection with influenza A virus (IAV, PR8 strain). **B,** Expression of *BLT1*. **C,** Expression of *BLT2*.

**Fig. S2 | LTB4 is still able to diminish NLRP3 inflammasome activation caused by IAV in BLT2*^-/-^* iBMDM**

**A,** WT and *BLT2^-/-^* iBMDM were primed with or without 100 nM LTB4 0.5 h, followed by infection with IAV (MOI = 10) for 16 h. Cell supernatants were collected for immunoblot analysis. (representative of three assays).

**Fig.S3 | LTB4 is still able to diminish NLRP3 inflammasome activation caused by IAV in *AIM2^-/-^* BMDMs.**

**A,** BMDMs isolated from WT and *AIM2^-/-^* mice were primed with or without 100 nM LTB4 0.5 h, followed by infection with IAV (MOI = 20) for 16 h. Cell supernatants were collected for immunoblot analysis. (representative of three assays).

**Fig. S4 | Bulk RNA-seq analysis of IAV infected BMDMs treated with LTB4.**

**A,** KEGG enrichment analysis. Top 10 pathways were shown. **B,** GSEA analysis showed the upregulation of cAMP mediated pathway.

**Fig. S5 | LTB4 is still able to increase the level of cAMP in** ***BLT2^-/-^* iBMDM.**

**A,** cAMP induction in WT and *BLT2^-/-^* iBMDM primed with or without 20 nM, 100 nM LTB4 0.5 h, followed by infection with IAV (MOI = 10) for 8 h. The concentration of cAMP in cells was quantified using ELISA. (representative of three assays).

**Statistics:** mean ± SD; two-way ANOVA (A); *p < 0.05, **p < 0.01, ***p < 0.001 and ****p < 0.0001.
