# Supplementary figures and images for: The LTB4-BLT1 axis attenuates influenza-induced lung inflammation by suppressing NLRP3 activation
Source: Cell Death Discov. 2025 Apr 6;11:148. doi: 10.1038/s41420-025-02450-8 (PMC11973165; doi:10.1038/s41420-025-02450-8)

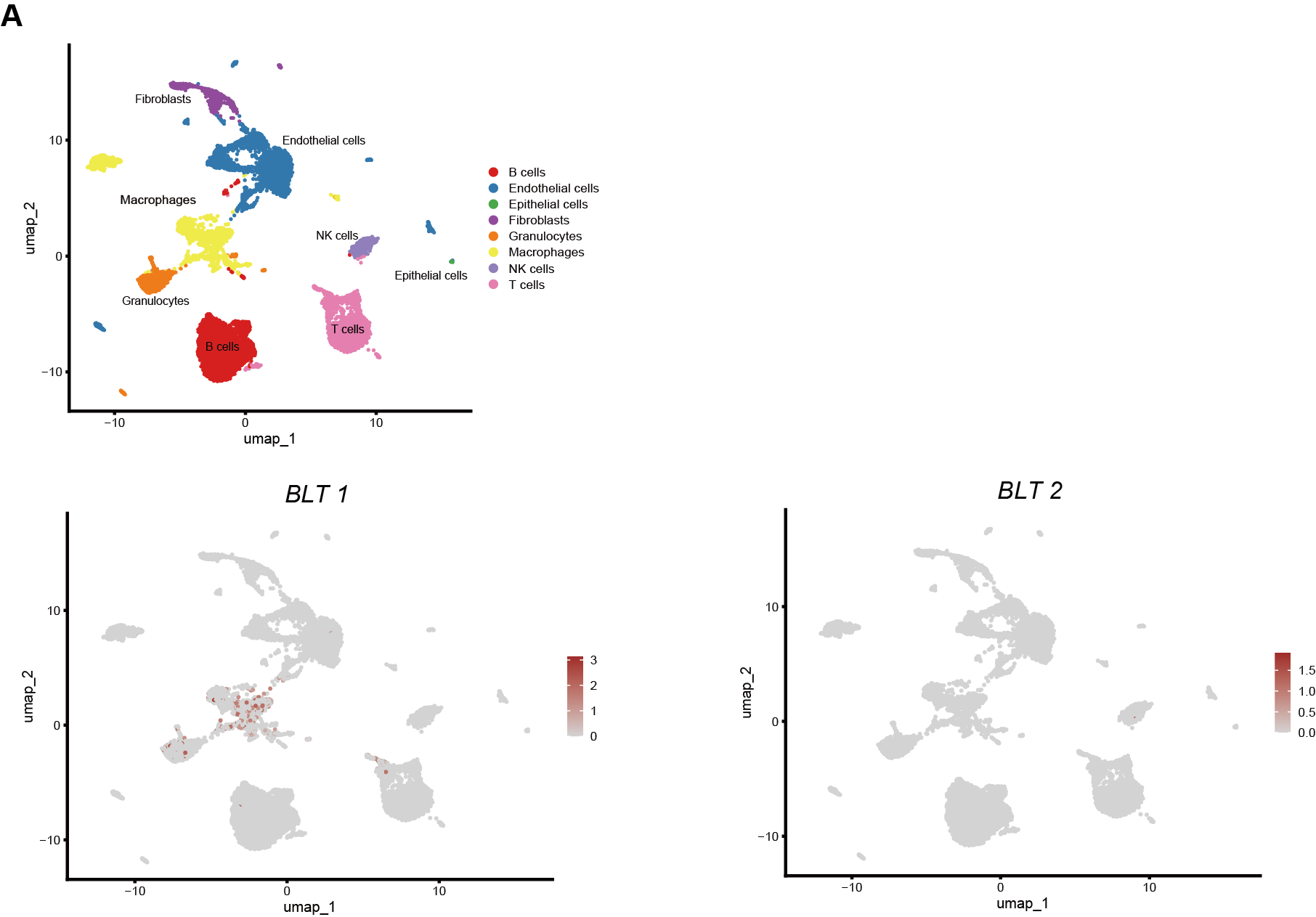

Supplement: Supplementary file 2 — Figure S1 [file 41420_2025_2450_MOESM2_ESM.png]

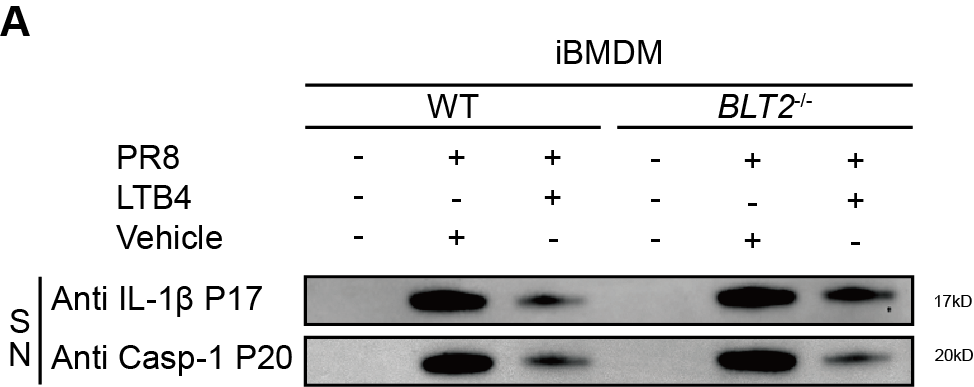

Supplement: Supplementary file 3 — Figure S2 [file 41420_2025_2450_MOESM3_ESM.png]

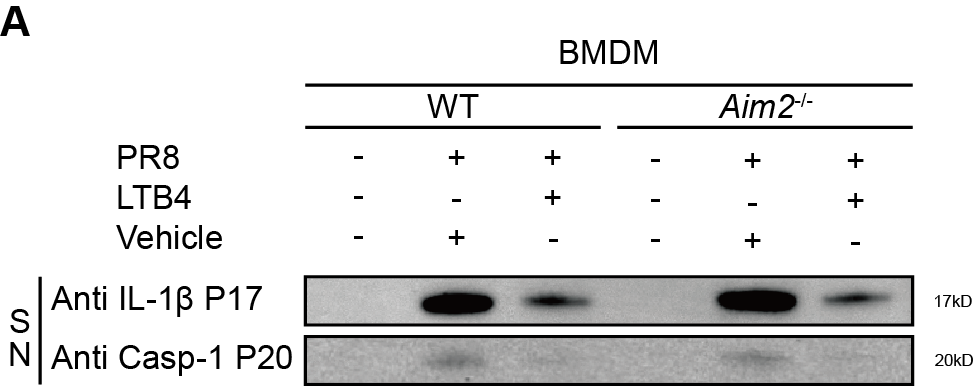

Supplement: Supplementary file 4 — Figure S3 [file 41420_2025_2450_MOESM4_ESM.png]

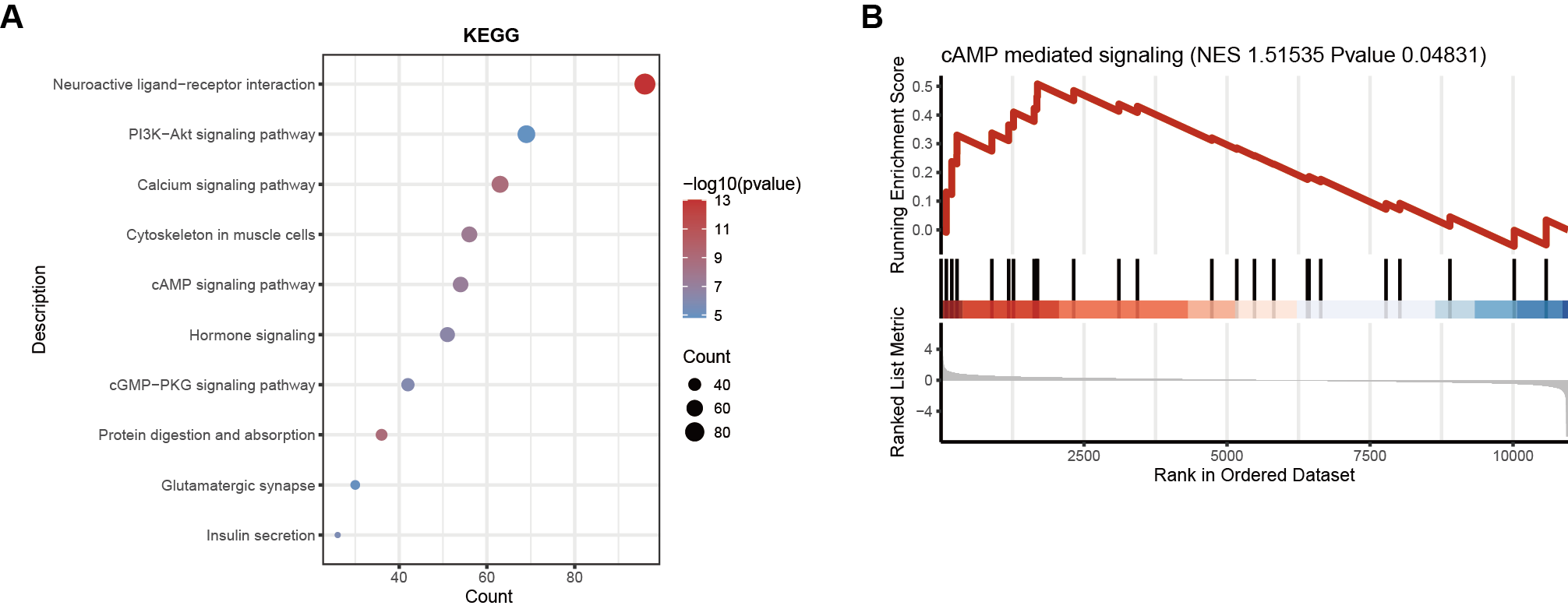

Supplement: Supplementary file 5 — Figure S4 [file 41420_2025_2450_MOESM5_ESM.png]

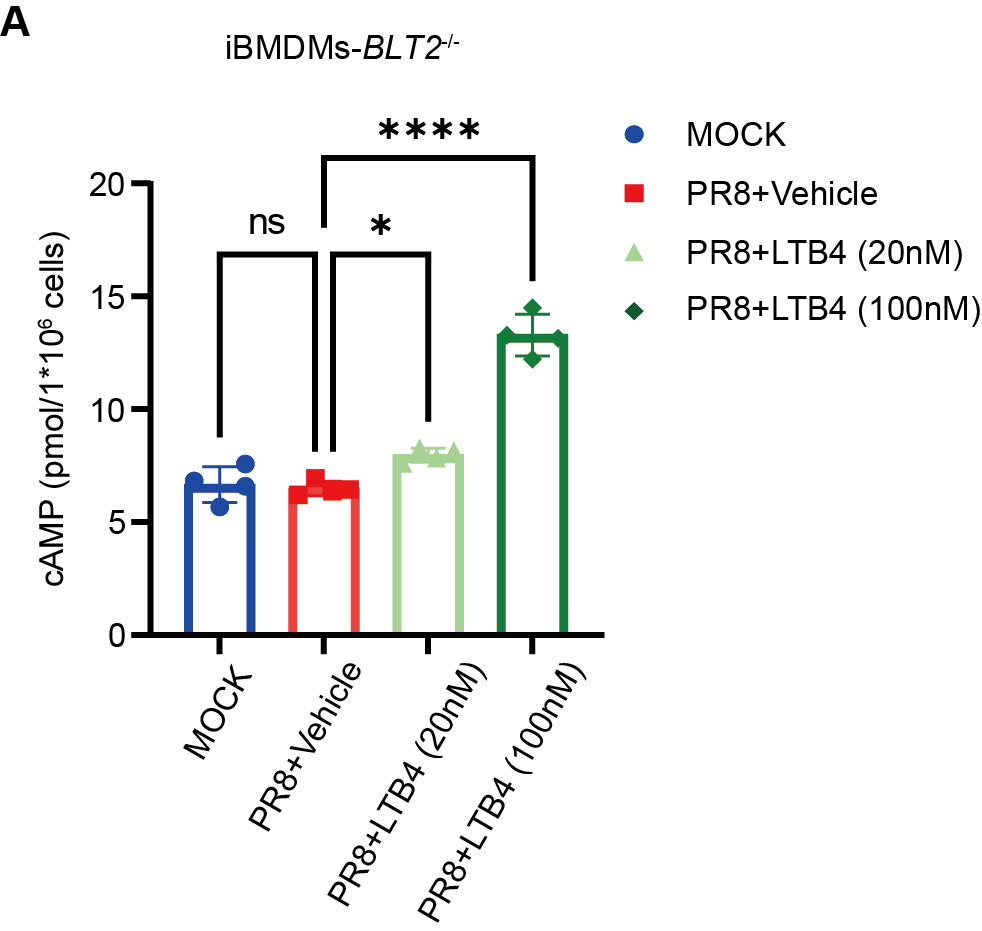

Supplement: Supplementary file 6 — Figure S5 [file 41420_2025_2450_MOESM6_ESM.png]
