# Supplementary material for: The LTB4-BLT1 axis attenuates influenza-induced lung inflammation by suppressing NLRP3 activation
Source: Cell Death Discov. 2025 Apr 6;11:148. doi: 10.1038/s41420-025-02450-8 (PMC11973165; doi:10.1038/s41420-025-02450-8)
Supplement: Supplementary file 7 — Author Contribution Statement [file 41420_2025_2450_MOESM7_ESM.docx]

**Author contribution**

B.C. and H.L. supervised the study and edited the paper. C.W. designed and performed the *in vitro* and *in vivo* experiments, analyzed and interpreted the data, and drafted the paper. Y.X. and Y.Z. participated in *in vitro* experiments and data analysis. L.H. and C.L. participated in animal experiments. All authors approved the final manuscript.
